# Case Report: Efficacy of Ninjin'yoeito Treatment for Idiopathic Pulmonary Fibrosis

**DOI:** 10.3389/fnut.2021.548076

**Published:** 2021-04-29

**Authors:** Hisako Kushima, Hiroshi Ishii, Masaki Fujita

**Affiliations:** ^1^Department of Respiratory Medicine, Fukuoka University Hospital, Fukuoka, Japan; ^2^Department of Respiratory Medicine, Fukuoka University Chikushi Hospital, Chikushino, Japan

**Keywords:** idiopathic pulmonary fibrosis, loss of appetite, malaise, ninjin'yoeito, Kampo medicine

## Abstract

Ninjin'yoeito, a Kampo prescription, was administered to two patients with idiopathic pulmonary fibrosis (IPF) over a period of 12 weeks to improve loss of appetite and lassitude. In Case 1, improvements were observed in appetite, lassitude, and breathlessness, as well as increases or increasing tendencies in body weight, blood albumin level, and hemoglobin (Hb) level. Case 2 showed no changes in appetite but improvements in lassitude and no deterioration of breathlessness. His body weight and his blood albumin and Hb levels increased or showed increasing trends. In both cases, a trend for improvement of respiratory function was observed. In summary, ninjin'yoeito trended to improve the subjective symptoms and nutritional status of a patient with pulmonary fibrosis.

## Introduction

Idiopathic pulmonary fibrosis (IPF) is a chronic and progressive disease in which advanced fibrogenesis results in irreversible honeycomb lungs. Antifibrotic agents can be used to treat IPF but the efficacy is limited. The mean lifespan after diagnosis of IPF is 3–5 years; and the mean lifespan after exacerbation of IPF is 2 months or less. IPF is associated with a very high mortality rate (1). Accompanying the progression of restrictive ventilatory defect, the IPF patient may complain of subjective symptoms, such as, exertional dyspnea, dry cough, weight loss, malaise, and/or fatigability. Since these symptoms significantly reduce patients' quality of life, we decided to administer ninjin'yoeito as an available medicine for malaise under universal health coverage in Japan. This report describes the administration of ninjin'yoeito to 2 IPF patients who had experienced loss of appetite and malaise, and a clear therapeutic response was observed.

## Case Report

**Figure 1:**
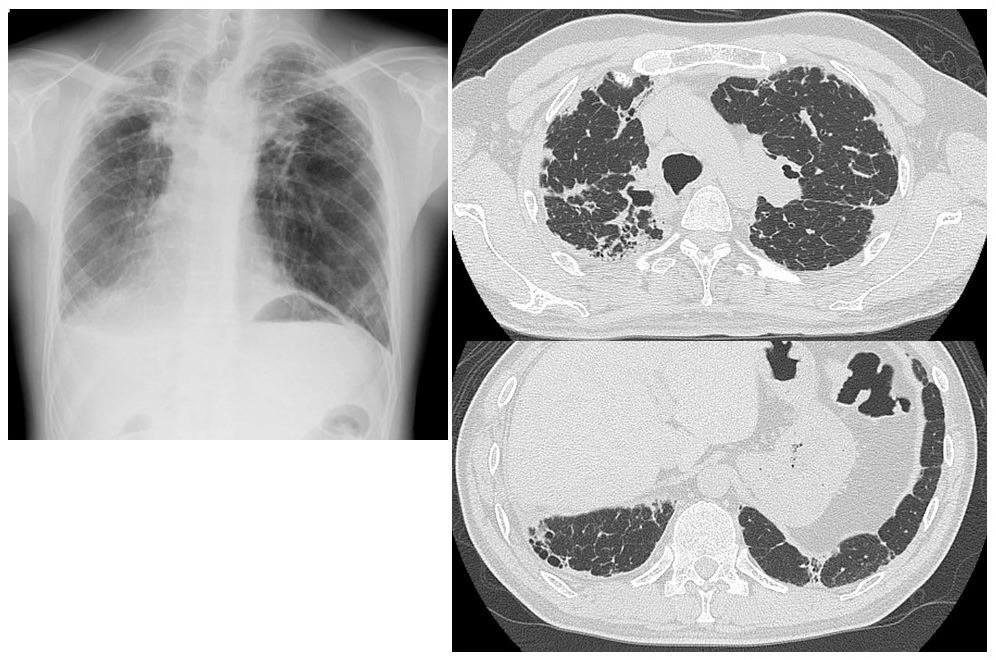
Chest radiograph and CT scans of Case 1 showing elevation of bilateral hilar opacities and subpleural reticular opacities in the bilateral lung fields.

Case 1 was a 59-year-old male with a history of rhabodomyolysis. The patient was referred to our hospital because of gradually progressive dyspnea on exertion. His height was 155.5 cm, the body weight was 51.8 kg and the percentage of oxygen saturation (SpO2) was 95%. His respiratory test results are shown in Table 1. Chest computed tomography (CT) demonstrated subpleural fibrotic opacities with traction bronchiectasis in the upper and lower lung fields. He was clinically diagnosed as IPF with pleuroparenchymal fibroelastosis-like lesions based on the guidelines (2) (Figure 1). He was treated with nintedanib ethanesulfonate and 7.5 g (divided into two doses) of ninjin'yoeito. The appetite score [Simplified Nutritional Appetite Questionnaire, SNAQ (3, 4)] showed improvement, increasing from 15 before administration to 16 after both 4 and 12 weeks of administration (SNAQ consists of four items related to appetite. All questions are scored from 1 to 5 points, with a total score ranging from 4 to 20 points. The lower the score, the poorer the appetite). Similarly, the lassitude score [Chalder Fatigue Scale, CFS (5, 6)] improved from six before administration to four after 4 and 12 weeks of administration (CFS consists of 11 items related to physical and mental fatigue. All questions are scored from 0 to 3 points, with a total score from 0 to 33 points. The higher the score, the stronger the fatigue). The breathlessness score [Modified Medical Research Council dyspnea scale, mMRC (7)] also improved from two before administration to one after both 4 and 12 weeks of administration (mMRC scale uses a 5-grade scale, with Grade 0 representing almost no breathlessness and Grade 4 representing severely impaired activities of daily living due to breathlessness. The higher the grade, the stronger the breathlessness). Body weight increased from 51.8 kg before administration to 54.5 and 55.2 kg after 4 and 12 weeks of administration, respectively. The blood albumin level increased from 3.6 g/dL before administration to 4.5 g/dL after both 4 and 12 weeks of administration, and the Hb level showed an increasing trend, changing from 14.9 g/dL before administration to 15.6 and 15.4 g/dL after 4 and 12 weeks of administration, respectively. The CONUT scores were zero before and 12 weeks after ninjin'yoeito administration. A trend for improvement was also observed with respect to respiratory function test results. Serum KL-6 decreased from 499 to 481 U/ml. LDH increased from 162 to 181 IU/L (Table 1). There was no occurrence of transaminitis during the treatment. He had an increase in walking distance from 383 to 440 m in a 6-min walk test (6MWT) 12 weeks after administration. The value of the lowest SpO2 at 6MWT remained unchanged at ~90% for 12 weeks. The CT finding did not show significant changes before and after treatment with ninjin'yoeito.

**Table 1:** The results of pulmonary function tests and biomarkers before and after ninjin'yoeito treatment in Case 1.

	**Before**	**After 12 weeks of**
	**administration**	**administration**
Vital capacity (VC) (L)	2.18	2.45
%Vital capacity (%VC)	63.2	72.5
Forced vital capacity (FVC) (L)	2.14	2.42
Forced expiratory volume 1.0 (FEV1) (L)	1.86	2.02
Forced expiratory volume 1.0% (FEV1%)	86.9	83.5
KL-6 (U/ml)	499	481
LDH (IU/L)	162	181

**Figure 2:**
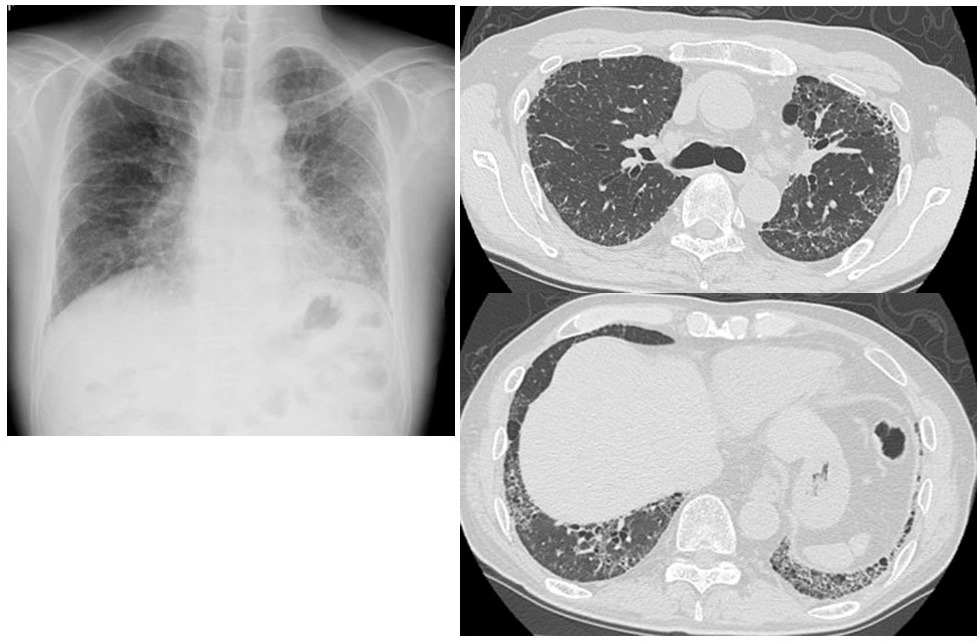
Chest radiograph and CT scans of Case 2 showing reticular opacities, traction bronchiectasis, and some ground-glass attenuation in the bilateral lung fields.

Case 2, a 59-year-old male was referred to our hospital for respiratory failure and was clinically diagnosed as IPF based on the guidelines [Raghu et al. (2)] (Figure 2).

His height was 167.5 cm, the body weight was 50.2 kg and the SpO2 was 90%. His respiratory test results are shown in Table 2. He was treated with nintedanib ethanesulfonate and 7.5 g (divided into 2 daily doses) of ninjin'yoeito. CFS decreased from 27 before administration to 16 and 24 after 4 and 12 weeks of administration, respectively, showing improvement. The mMRC scale was three before administration and 2 and 3 after 4 and 12 weeks of administration, respectively, showing no deterioration. The SNAQ score was 14 before administration and after 4 and 12 weeks of administration, showing no change. Body weight increased from 50.2 kg before administration to 50.4 and 51.9 kg after 4 and 12 weeks of administration, respectively. The blood albumin level was 4.1 g/dL before administration to 4.1 and 4.3 g/dL after 4 and 12 weeks of administration, respectively, showing an increasing trend. The Hb level increased from 12.8 g/dL before administration to 13.7 and 14.3 g/dL after 4 and 12 weeks of administration, respectively. The CONUT score could not be measured because the serum cholesterol levels were not measured. The levels of albumin and lymphocyte counts were normal throughout the treatment period. A trend for improvement was also observed with respect to respiratory function. Serum KL-6 decreased slightly from 496 to 492 U/ml. LDH decreased from 201 to 196 IU/L (Table 2). There was no occurrence of transaminitis during the treatment. A 6MWT after 14 weeks of administration showed improvement in the lowest SpO2 (from 87 to 91%) while the walking distance decreased from 360 to 300 m. The CT finding did not show significant changes before and after treatment with ninjin'yoeito.

**Table 2:** The results of pulmonary function tests and biomarkers before and after ninjin'yoeito treatment in Case 2.

	**Before**	**After 12 weeks of**
	**administration**	**administration**
Vital capacity (VC) (L)	1.65	1.90
%Vital capacity (%VC)	42.3	48.7
Forced vital capacity (FVC) (L)	1.62	1.83
Forced expiratory volume 1.0 (FEV1) (L)	1.19	1.43
Forced expiratory volume 1.0% (FEV1%)	73.5	78.1
KL-6 (U/ml)	496	492
LDH (IU/L)	201	196

## Discussion

Ninjin'yoeito was administered to patients with IPF in this study. The treatment improved subjective symptoms such as, loss of appetite, malaise, and breathlessness, as well as scores related to nutritional status, including body weight and blood albumin and Hb levels. In a basic pharmacological study using a bleomycin-induced pulmonary fibrosis model, ninjin'yoeito was reported to have suppressed decreases in food intake and body weight, atrophy of the gastrocnemius, and expression of inflammatory cytokines in the lungs (8). Regarding the mechanism by which it improves loss of appetite, activation of ghrelin-responsive, and unresponsive neuropeptide Y neurons in the arcuate nucleus of the hypothalamus (9) and actions mediated by dopamine D_2_ receptors (10) have been reported.

Ninjin'yoeito was administered to the two patients described herein to improve subjective symptoms, such as, loss of appetite and malaise. In addition to these improvements, trends for improvement were also observed with respect to respiratory function after 12 weeks of administration. Ninjin'yoeito is a Kampo prescription consisting of 12 crude drugs. Unlike hochuekkito and juzentaihoto, which are Kampo prescriptions that are similarly used to improve lassitude and malaise, ninjin'yoeito contains schisandra fruit, polygala root, and citrus unshiu peel, which all act on the respiratory system. A component of schisandra fruit, α-cubebenoate, has been reported to suppress accumulation of eosinophils, macrophages, and lymphocytes in the pulmonary alveoli in a bronchial asthma model and inhibited Th2 cytokine and TGF-β1 in pulmonary tissue (11). In a pneumonitis model, ninjin'yoeito was shown to suppress lung injury and death as well as production of nitric oxide in the serum (12). Pirfenidone is a therapeutic agent for IPF and has been shown to have an antifibrotic action in models of pulmonary fibrosis and hepatic cirrhosis (13, 14). Similarly, in a hepatic fibrogenesis model, ninjin'yoeito was found to suppress increases in the hepatic levels of hydroxyproline, which is an index of total collagen, as well as the production of cytokines that induce fibrogenesis (TGF-β1 and IL-13) (15). Investigation of additional cases will help to clarify the effect of ninjin'yoeito for the treatment of respiratory diseases. We believe that our study makes a significant contribution to the literature because the findings showed that NYT can improve fatigue and contribute to maintaining quality of life in IP patients.

## Data Availability Statement

The original contributions presented in the study are included in the article/supplementary material, further inquiries can be directed to the corresponding author/s.

## Ethics Statement

The studies involving human participants were reviewed and approved by Fukuoka University Hospital Ethics Committee. The patients/participants provided their written informed consent to participate in this study. Written informed consent was obtained from the individual(s) for the publication of any potentially identifiable images or data included in this article.

## Author Contributions

HK conducted the study and wrote the manuscript. HI and MF instructed the study. All authors contributed to the article and approved the submitted version.

## Conflict of Interest

The authors declare that the research was conducted in the absence of any commercial or financial relationships that could be construed as a potential conflict of interest.
